# Extracts of *Portulaca oleracea* and *Patrinia scabiosaefolia* relieve ultraviolet B-induced skin injury in solar dermatitis mice via inhibiting IL-17/CCL2 pathway and oxidative stress

**DOI:** 10.7150/ijms.106289

**Published:** 2025-01-21

**Authors:** Bowei Li, Jingyuan Yang, Zhixing Wang, Mingzhu He, Xujing Chen, Yixuan Chen, Baoying Shen, Junru Chen, Chunqi Yang, Tian Li, Chengcai Lai, Yue Gao, Hong Cai

**Affiliations:** 1 Chinese PLA Medical School, Chinese PLA General Hospital, Beijing 100853, China.; 2 Department of Dermatology, Air Force Medical Center, PLA, Beijing 100142, China.; 3 Department of Pharmaceutical Sciences, Beijing Institute of Radiation Medicine, Beijing 100850, China.; 4 Tianjin Key Laboratory of Acute Abdomen Disease-Associated Organ Injury and ITCWM Repair, Institute of Integrative Medicine of Acute Abdominal Diseases, Tianjin Nankai Hospital, Tianjin Medical University, 8 Changjiang Avenue, Tianjin 300100, China.; 5 State Key Laboratory of Kidney Diseases, Chinese PLA General Hospital, Beijing 100853, China.; 6 Medical College, Qinghai University, Xining 810016, China.

**Keywords:** Solar dermatitis, *Portulaca oleracea*, *Patrinia scabiosifolia*, UPLC-HRMS, IL-17/CCL2 pathway, Oxidative stress

## Abstract

**Introduction:** Solar dermatitis, a condition triggered by excessive exposure to ultraviolet B (UVB) radiation, results in inflammatory skin damage marked by erythema, edema, and epidermal injury. Portulaca oleracea (PO) and Patrinia scabiosaefolia (PS) have been traditionally used in dermatological treatments, though their mechanistic pathways in UVB-induced skin injury are not fully understood.

**Methods:** In this study, a mouse model of UVB-induced solar dermatitis was employed to evaluate the therapeutic potential of combined PO and PS (POPS) extracts. After UVB irradiation, POPS extracts were administered, and their bioactive compounds were identified through ultra-performance liquid chromatography-mass spectrometry (UPLC-MS). Network pharmacology, molecular docking, and pathway analysis were performed to identify key targets, focusing on the IL-17/CCL2 pathway and oxidative stress reduction.

**Results:** Treatment with POPS extracts significantly diminished UVB-induced inflammation, erythema, and epidermal thickening in a dose-dependent manner. Network pharmacology and docking studies identified curvularin and olmesartan medoxomil as bioactive components with high affinity for IL-17 and IL-17RA targets, modulating the IL-17/CCL2 axis. *In vivo* experiments demonstrated that POPS extracts suppressed the expression of IL-17 and CCL2, reduced macrophage infiltration, and alleviated oxidative stress, effectively mitigating the symptoms of solar dermatitis.

**Conclusion:** This study provides insight into the anti-inflammatory and protective properties of POPS extracts in solar dermatitis, highlighting their potential as a treatment through IL-17/CCL2 pathway modulation and oxidative stress reduction.

## Introduction

Excessive exposure to ultraviolet (UV) radiation, particularly ultraviolet B (UVB) rays, is a major environmental factor that contributes to the development of solar dermatitis, a prevalent skin condition marked by acute inflammatory responses such as erythema, edema, and epidermal thickening 1. The increasing intensity of UV radiation, driven by ozone depletion and environmental pollution, has led to an increase in cases of solar dermatitis globally, particularly in regions with high sunlight exposure 2, 3. Current treatment strategies primarily involve the use of corticosteroids and nonsteroidal anti-inflammatory drugs (NSAIDs), which are associated with adverse effects, including skin atrophy, capillary dilation, and the potential for long-term dependency. Consequently, there is a pressing demand for the development of safer and more effective treatment alternatives.

In traditional Chinese medicine (TCM), solar dermatitis is viewed as a condition arising from internal heat, dampness, and toxin accumulation and is often exacerbated by external environmental heat. TCM formulations that focus on clearing heat, detoxifying the body, and resolving dampness are frequently used in clinical settings to manage this condition, providing therapeutic benefits with fewer side effects. One such formulation, XianCaoHeJi (XCHJ), which consists of *Portulaca oleracea* (PO) and *Patrinia scabiosaefolia* (PS), has been widely employed. Oxidative stress is a cellular stress 4, 5. *Portulaca oleracea*, a globally recognized medicinal plant, is well-documented for its anti-inflammatory, antioxidant, and antibacterial properties 6, 7, whereas *Patrinia scabiosaefolia* has been employed in TCM for centuries to treat various inflammatory disorders 8, 9. Clinically, the topical application of a decoction made from PO and PS has demonstrated promising results in treating solar dermatitis; however, the exact pharmacological mechanisms underlying its efficacy remain unclear.

Recent advancements in phytochemical analysis and network pharmacology have provided novel insights into the bioactive compounds in TCM and their mechanisms of action. Modern studies indicate that the constituents of POPS possess notable anti-inflammatory, immunomodulatory, and antioxidative properties, positioning them as promising candidates for the treatment of UV-induced skin damage 10, 11. Research has demonstrated that PO can lower the levels of pro-inflammatory cytokines, including IL-β and IL-6, while simultaneously increasing the level of IL-10, thereby suppressing LPS-induced pulmonary inflammation 12, 13. Iridoid components in PS have been shown to inhibit the production of nitric oxide (NO) and reactive oxygen species (ROS) 14, while the compounds patrinoside and patrinoside A mitigate insulin resistance by inhibiting the NF-κB and MAPK signaling pathways as well as oxidative stress 15. The IL-17 signaling pathway, which mediates inflammatory responses, along with oxidative stress, plays a crucial role in the pathogenesis of solar dermatitis by driving the recruitment of immune cells and exacerbating tissue damage 16, 17. Consequently, inhibition of these pathways may represent an effective therapeutic strategy for alleviating the symptoms of solar dermatitis.

The inherent complexity of Traditional Chinese Medicine (TCM) presents significant challenges to pharmacodynamic research, necessitating the systematic identification of its components as a crucial preliminary step. In recent years, ultra-performance liquid chromatography coupled with high-resolution mass spectrometry (UPLC-HRMS) has become the leading methodology for analyzing TCM components due to its exceptional efficiency, sensitivity, and selectivity. This study utilized UPLC-HRMS to precisely identify the chemical constituents within samples, successfully determining the structures of 12 components through database comparison. By incorporating network pharmacology analysis, this research offers a novel perspective on TCM mechanisms from a "drug-target-disease" standpoint. The pharmacologically active components of POPS extracts demonstrate significant efficacy in the prevention and treatment of solar dermatitis. This study elucidated that POPS confers protection against UV-induced skin damage in mice by inhibiting the IL-17/CCL2 signaling pathway and mitigating oxidative stress, underscoring its potential as an innovative therapeutic strategy with promising clinical applications.

## Materials and Methods

### Chemicals and reagents

PO herbal materials were purchased from Beijing Bencao Fangyuan Pharmaceutical Group Co., Ltd. (Beijing, China). PS herbal materials were obtained from Jiangsu Chuan Cheng Chinese Medicine Co., Ltd. (Jiangsu, China), and hydrocortisone butyrate cream (HBC) was procured from Tianjin Jinyao Pharmaceutical Co., Ltd. (Tianjin, China). Poloxamer 188 was obtained from Aladdin (Shanghai, China), and poloxamer 407 was obtained from BASF (Germany). Rabbit anti-mouse MMP-2 and MMP-9 antibodies were purchased from Abcam (Cambridge, UK). Rabbit anti-mouse β-actin, p-p38MAPK, p38MAPK, p-p42MAPK, p42MAPK, c-JUN, IL-1β, IL-6, TNF-α, and F4/80 antibodies were acquired from Cell Signaling Technology (Massachusetts, USA). Mouse anti-mouse IL-17 and rabbit anti-mouse Act1 and CCL2 antibodies were obtained from Proteintech (Wuhan, China). Rabbit anti-mouse IL-17RA antibody was sourced from ABclonal (Wuhan, China). MDA, LPO, and GSH ELISA kits were obtained from Meimian (Jiangsu, China). A dihydroethidium and cellular senescence β-galactosidase staining kit was procured from Beyotime (Shanghai, China). A TUNEL assay kit was obtained from Roche (Basel, Switzerland). One-step gDNA removal, cDNA synthesis supermix and SYBR Green qPCR supermix were obtained from TransGen (Beijing, China). All experiments were conducted in accordance with the manufacturers' instructions.

### Solar dermatitis mouse model

Prior to the experiment, healthy male SPF-grade C57BL/6J mice underwent a seven-day acclimatization period. The mice were housed under a 12-hour light‒dark cycle with ad libitum access to food. The animal center maintained the environmental temperature at 22 ± 2°C and the relative humidity at 50 ± 10%. Twenty-four hours before modeling, a 4 × 2 cm^2^ area of dorsal hair was shaved in preparation for UVR treatment of 6- to 8-week-old C57BL/6J mice. These mice were provided by Beijing Vital River Laboratory Animal Technology Co., Ltd., (license number SCXK [Jing] 2021-0006). Forty C57BL/6J mice were randomly divided into five groups to undergo irradiation for various durations (0, 15, 30, 45, or 60 minutes), with 8 mice per group **(Figure S1D)**. A UV lamp (ROYAL PHILIPS, Netherlands) with a peak wavelength of 311 nm and a dose of 0.45 mW/cm^2^ that was positioned 20 cm away was used to irradiate the shaved areas to establish the solar dermatitis model **(Figures S1A-C)**. The severity of dermatitis was assessed at 24, 48, and 72 h after UVB irradiation. Skin manifestations such as erythema, edema, and skin destruction were scored as 0 (absent), 1 (mild), 2 (moderate), or 3 (severe) to evaluate the extent of UV-induced skin damage 18. At 72 h, skin tissue samples were collected for hematoxylin and eosin (HE) staining to observe histopathological changes and epidermal thickness, as well as Masson's trichrome staining to examine the collagen fiber proliferation in the dermis.

### Preparation of POPS extracts and temperature-sensitive *in situ* hydrogels (ISGs)

For the PO extraction, 100 g of PO was soaked in 1000 mL of distilled water, heated, and filtered. This process was repeated, and the solution was concentrated to 1000 mL, lyophilized to yield 27.4 g of powder, and stored at -20°C. The same method was used for PS, yielding 9.3 g of powder. For ISGs, poloxamers P407 and P188 were mixed with water **(Figure S2A)** and incubated at 4°C 19. For POPS ISGs, 0.66 g of PO aqueous extract and 0.15 g of PS aqueous extract were accurately measured and placed into three 50-mL centrifuge tubes. Then, 10, 20, or 40 ml of the thermosensitive hydrogels were added to achieve concentrations of 40%, 20%, and 10%, respectively **(Figure S2B)**. After thorough mixing with a vortex, the mixture was stored at 4°C.

### Animals and treatments

In this study, 42 healthy male SPF-grade C57BL/6J mice were randomly assigned to seven groups: the control group, the model group, the positive control drug (HBC) group, the thermosensitive hydrogel group, and the 10%, 20%, and 40% POPS treatment groups. Twenty-four hours prior to UV irradiation, a 4 × 2 cm^2^ area of skin on the dorsum of each mouse was shaved. Except for the control group, all other groups were exposed to 810 mJ/cm^2^ UVB radiation. The mice in the treatment groups were topically administered 500 μL of extracts of POPS thermosensitive ISGs at concentrations of 10%, 20%, or 40% twice daily. The positive control drug group and the thermosensitive hydrogel group received the same dose at the same frequency. Changes in the mice's appearance were observed and recorded at 24, 48, and 72 h after UV irradiation **(Figure 2A)**. After 72 hours, skin tissue samples were collected from the mice for ELISA, Western blotting, qRT‒PCR, histological analysis, immunohistochemistry, and immunofluorescence staining. This study adhered to the guidelines of the AAALAC.

### Cell culture and treatment

Human macrophages (FH-H096) and human skin fibroblasts (HSF, FH0189) were obtained from FuHeng Biology (Shanghai, China). HSF cells were cultured in DMEM/F12 medium supplemented with 15% fetal bovine serum (FBS) and 1% penicillin‒streptomycin, while macrophages were cultured in specialized complete macrophage medium (PY-H096) at 37°C in a 5% CO₂ atmosphere. The heat-sterilized POPS lyophilized powder was dissolved in sterile phosphate-buffered saline (PBS) to prepare solutions of 5, 10, and 20 μg/mL, which were then filtered through a 0.22-μm membrane to ensure sterility to ensure sterility. Macrophages were seeded into 6-well plates at a density of 1×10⁶ cells per well. Upon reaching 80-90% confluence, the medium was aspirated, and the cells were washed twice with PBS. The macrophages were then treated with 5, 10, or 20 μg/mL POPS solutions for 8 hours. After treatment, the cells were washed with PBS to remove residual compounds. The macrophages were exposed to UVB radiation at a dose of 50 mJ/cm^2^. Following UVB treatment, fresh medium was added, and the cells were cultured for an additional 24 hours. After 24 hours, the macrophage culture supernatants were collected, centrifuged at 3000 rpm for 10 min to remove cell debris, and stored at -80°C for future use. HSF cells were seeded into 6-well plates and cultured until they reached 80-90% confluence. The culture medium was then replaced with the macrophage supernatants, and the cells were cultured for another 24 hours. Finally, senescence-associated β-galactosidase (SA-β-gal) was performed to evaluate cellular senescence.

### Extraction of pharmacological components from the skin tissue of the treatment group

For the POPS extraction, 600 μL of the sample was mixed with 400 μL of pure methanol and vortexed for 10 seconds, Subsequently, 200 μL of the mixture was then added to 200 μL of a 40% methanol solution, vortexed again, and centrifuged at 16,000 × g at 4°C for 15 minutes. The supernatant was collected.

For the extraction of 40% POPS from tissue, 100 mg of skin tissue was mixed with 300 μL of water and ground using a cryogenic mill. Next, 700 μL of methanol was added. The sample was ultrasonicated for 20 minutes in a cold water bath and then centrifuged at 16,000 × g at 4°C for 15 minutes. Following centrifugation, the supernatant was vacuum dried. Then, 100 μL of 40% methanol solution was added, the mixture was vortexed and centrifuged, and the supernatant was collected.

For the combined control and POPS extraction, 100 mg of control tissue was ground with 300 μL of water, and 700 μL of methanol or herbal solution was then added. The sample was ultrasonicated for 20 minutes, centrifuged at 16,000 × g at 4°C for 15 minutes, and vacuum-dried. Afterward, 80 μL of 40% methanol solution was added. The mixture was vortexed and centrifuged, and the supernatant was collected.

### Ultrahigh-performance liquid chromatography (UPLC) combined with high-resolution mass spectrometry (HRMS)

POPS extracts were analyzed using UHPLC coupled with a Q Exactive HFX mass spectrometer. A gradient elution program with a flow rate of 0.3 mL/min was employed. Mass spectra were acquired in both positive and negative ionization modes, and compounds were identified by comparing their mass, isotopic distributions, and MS/MS spectra with reference databases.

### Network pharmacology analysis

We utilized the Swiss Target Prediction platform to identify the pharmacological components of POPS detected via UPLC-HRMS, along with their corresponding targets. We then identified potential targets associated with solar dermatitis from the GeneCards, PharmGkb, and OMIM databases, as depicted in **Figure 3C**. These targets were compared with the solar dermatitis-related gene set to identify common genes, which are illustrated in a Venn diagram. Subsequently, a "POPS-Pharmacological Compounds-Target Gene" network diagram was constructed using Cytoscape 3.8.0, and the shared genes were uploaded to the STRING database as gene symbols to generate a protein‒protein interaction (PPI) network. To further elucidate the biological implications of these genes, we conducted Gene Ontology (GO) functional enrichment and Kyoto Encyclopedia of Genes and Genomes (KEGG) pathway enrichment analyses via R 4.3.2. The GO analysis encompassed three domains, biological processes, cellular components, and molecular functions, with the top 10 items in each domain visualized based on a significance threshold of P < 0.05. Additionally, KEGG enrichment analysis identified the top 30 signaling pathways according to the criterion P < 0.05, providing insights into the molecular mechanisms through which POPS may exert its therapeutic effects on solar dermatitis.

### Molecular docking

The 2D structures of the small-molecule ligands were downloaded from the PubChem database andthen converted into 3D structures using ChemOffice. The core target protein structures were downloaded from the PDB database and preprocessed using PyMol 2.4.0 software to remove water molecules and separate the original ligands. Subsequently, hydrogenation and assignment of atom types were performed via AutoDock 1.5.6. Small-molecule structures were downloaded from the Protein Data Bank and saved in the mol2 format. Molecular docking of the core components was subsequently performed using AutoDock and Vina.

### Observed indicators

#### The general state

The mental state, fur condition, limb condition, activity levels, and dietary habits of the mice were monitored. Changes in their appearance were photographed every 24 hours, and scratching behavior was observed for 20 minutes following each observation period. Scratching behavior was scored on a scale of 0 to 3 based on duration, and the scores were summed to determine the level of itchiness 20.

#### Hematoxylin‒eosin (HE)

Skin samples* (*1 cm × 1 cm) were fixed in paraformaldehyde, processed, and stained with hematoxylin and eosin. Epidermal thickness was examined under a microscope and analyzed using Caseviewer software.

#### Masson's trichrome staining

The sections were dewaxed, rehydrated, and stained with iron hematoxylin, acid fuchsin, and aniline blue, followed by differentiation and dehydration. Microscopic observation and quantitative analysis were conducted using ImageJ software.

#### Immunohistochemistry

The tissue sections were dewaxed, rehydrated, subjected to antigen retrieval, blocked, and subsequently incubated with primary and secondary antibodies. DAB staining, hematoxylin counterstaining, and microscopy imaging were performed. Quantitative analysis was conducted using ImageJ software.

#### Immunofluorescence

The sections were dewaxed, rehydrated, and treated with proteinase K for antigen retrieval. Subsequently, TUNEL and DAPI staining were performed. Observations were made under a fluorescence microscope, and quantitative analysis was carried out using ImageJ software. ROS (dihydroethidium) staining was performed following the same protocol.

#### ELISA

Skin tissues (0.1 g) were homogenized in 0.9 mL of pre-cooled PBS and then centrifuged at 4°C at 5000 × g for 10 minutes. The supernatant was collected, and protein quantification was performed using a BCA assay kit. The levels of LPO, MDA, GSH, IL-1β, IL-6, and TNF-α were measured using the corresponding ELISA kits according to the manufacturers' instructions.

#### Western blotting

Proteins were extracted from skin tissue, separated by SDS‒PAGE, and transferred onto a PVDF membrane. After blocking, the membranes were incubated with primary and secondary antibodies, developed using a chemiluminescence reagent kit, and imaged. Band intensities were analyzed using ImageJ software.

#### Quantitative Real-time PCR (qRT‒PCR)

RNA was extracted from skin tissues, reverse-transcribed into cDNA, and amplified using SYBR Green qPCR Supermix. The amplification program was carried out on a CFX96 instrument, and primers were designed on the based on NCBI data (**Table 1**).

### Data analysis

All reported results represent the results of three or more independent experiments and are presented as means ± SDs. Data analysis and visualization were conducted using GraphPad Prism 8. When multiple independent groups were compared, one-way ANOVA followed by Tukey's post hoc test was applied to assess statistical significance. For comparisons involving two independent variables, we utilized two-way ANOVA. A p-value of less than 0.05 was considered to indicate statistical significance.

## Results and Discussion

### Protective effects of POPS extracts in a mouse model of solar dermatitis

**Figure 1:**
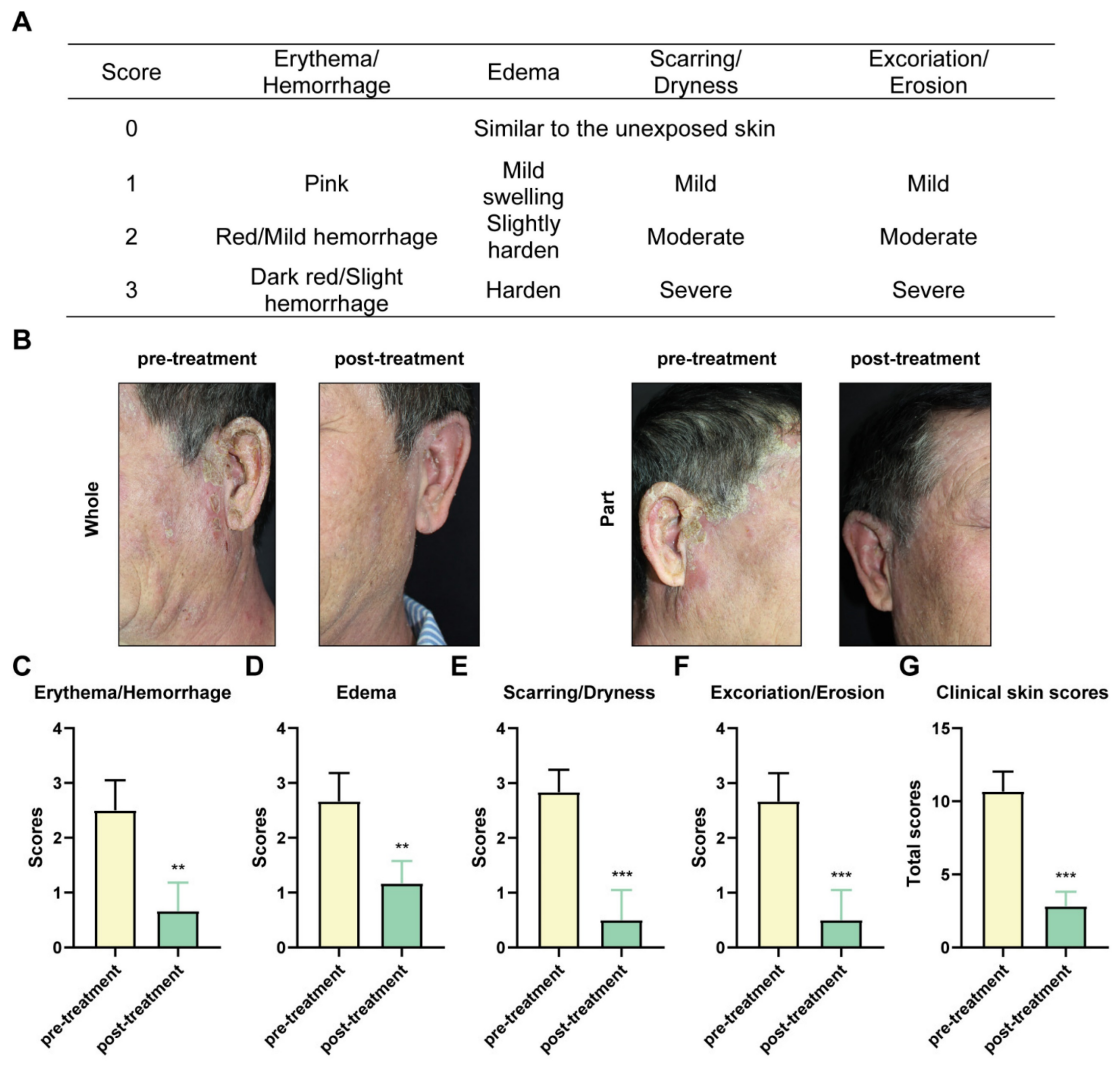
** The clinical efficacy of POPS in treating solar dermatitis is significant.** (A) Clinical skin scores for individuals with solar dermatitis. (B) Clinical features of patients with solar dermatitis before and after 20 days of twice-daily topical applications of POPS. Photodamage symptoms, including erythema/hemorrhage (C), edema (D), scarring/dryness (E), excoriation/erosion (F), and the total score (G), were assessed using a four-point Likert scale. Symptoms were significantly alleviated following treatment.

POPS extracts, characterized by their natural composition and minimal side effects, have demonstrated remarkable efficacy in treating solar dermatitis. In clinical practice, POPS is administered as a wet dressing applied twice daily to affected skin areas. In a representative case study, [R] following 20 days of POPS treatment, a 55-year-old patient showed significant improvement in erythema and bleeding symptoms, as evidenced by clinical skin score analysis **(Figure 1A)** 21. These results demonstrated that] the inflammatory response was effectively suppressed **(Figure 1B)**. The degree of skin edema showed marked improvement** (Figure 1C)**, presumably due to reduced vascular permeability and decreased inflammatory mediator production. Furthermore, clinical manifestations including scar formation, skin dryness, and stratum corneum desquamation showed substantial improvement **(Figures 1D-E)**, indicating progressive restoration of the skin barrier function and enhanced tissue regeneration. Of particular significance, the patient's overall clinical skin score exhibited a significant reduction post-treatment** (Figure 1G)**, confirming the reliability and sustainability of the therapeutic effect. Importantly, no adverse reactions were documented during the two-month follow-up period.

While these preliminary findings demonstrate POPS's therapeutic potential in solar dermatitis management, the underlying pharmacological mechanisms remain poorly understood. To address this knowledge gap, we developed an animal model of solar dermatitis to investigate its molecular mode of action. These experimental models utilize UV radiation to replicate human solar dermatitis symptoms, including skin erythema, inflammation, and cellular damage, enabling detailed study of disease pathogenesis 22. Through rigorous experimental control, we systematically evaluated and optimized UVB radiation dosage and exposure duration to establish a reproducible and clinically relevant animal model. [R] Our findings showed that radiation at 405 mJ/cm^2^ (15 minutes) induced erythema, keratinocyte hyperplasia, and epidermal thickening** (Figures S1E-H)**. At 810 mJ/cm^2^ (30 minutes), we observed more severe damage characterized by significant epidermal thickening and pathological collagen fiber proliferation** (Figure S1I)**. Higher doses (1215 and 1620 mJ/cm^2^) resulted in severe skin damage, characterized by indistinct epidermal structure, abnormal keratinocyte hyperplasia, disrupted dermal collagen fiber organization, and extensive inflammatory cell infiltration **(Figures S1I-K)**. Based on these observations, we selected 810 mJ/cm^2^ as the optimal dose for subsequent experiments, as its efficacy in replicating the clinical manifestations associated with severe second-degree sunburn, such as erythema, blistering, and burning sensations. This dosage induces damage to both the epidermis and dermis, eliciting a marked inflammatory response that closely mimics the pathology of severe sunburn. Furthermore, histopathological analysis has verified that this dose accurately reproduces the dermal damage characteristic of superficial second-degree burns 22, thereby establishing it as an optimal model for assessing the therapeutic efficacy of POPS extract in the treatment of severe skin injuries.

Following the establishment of an optimal animal model, we focused on developing the most effective POPS administration strategy. While topical application of POPS decoction has shown promising therapeutic outcomes in clinical settings, implementing this approach in preclinical models presents several technical challenges, particularly regarding dose consistency and skin contact duration. To overcome these limitations and enhance experimental reproducibility, we implemented thermosensitive hydrogels as an advanced drug delivery system.

**Figure 2:**
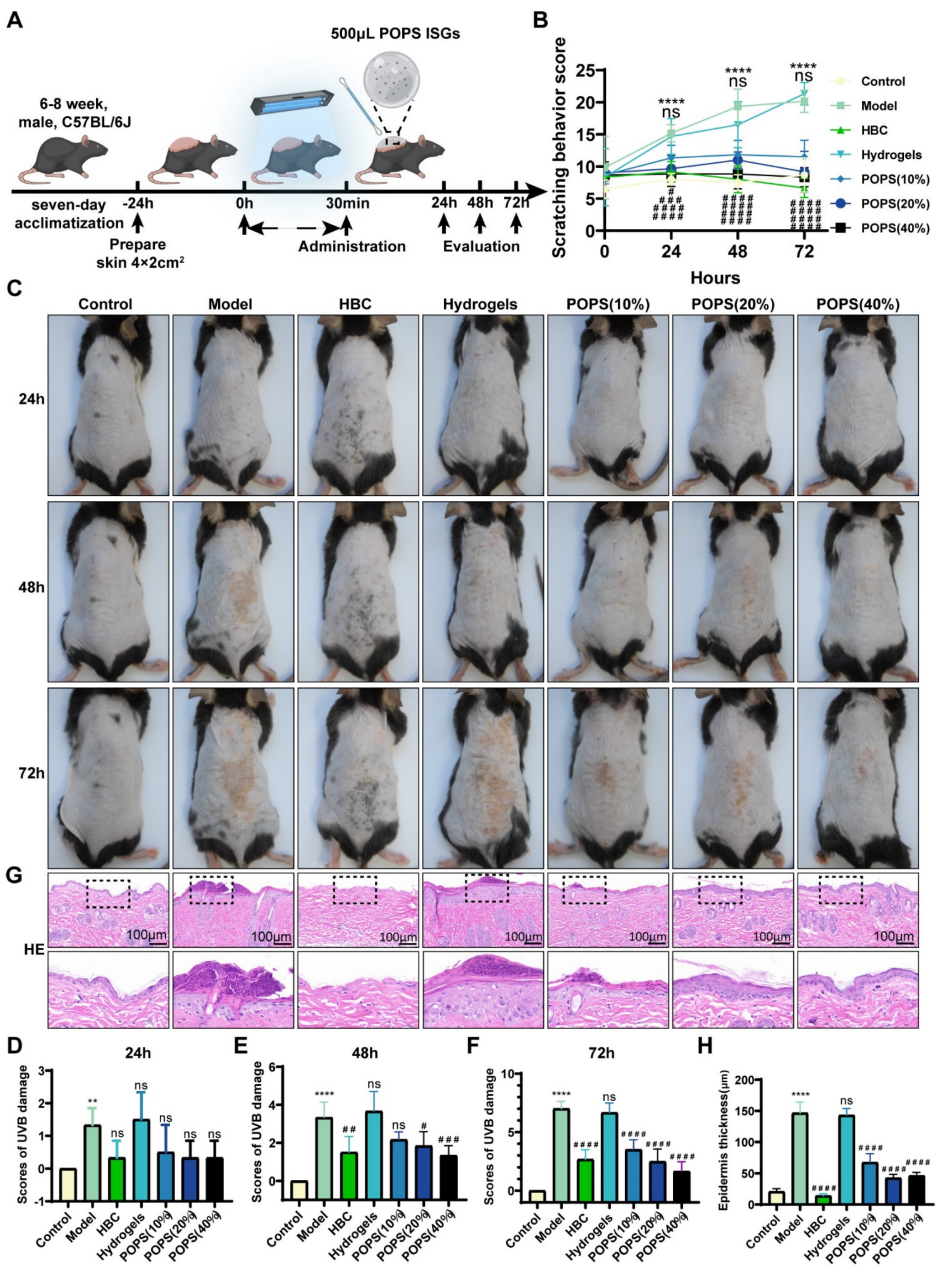
** Protective effects of POPS extracts in a mouse model of solar dermatitis.** (A) Schematic diagram illustrating the establishment of a solar dermatitis model in C57BL/6J mice using a 30-minute (810 mJ/cm^2^) UVB irradiation dose to validate the efficacy of POPS extracts. (B) Quantitative analysis of scratching behavior in mice during the treatment period, with scoring details described in the "Observed Indicators" section. (C) Representative images of skin appearance in groups of mice exposed to UVB for 24, 48, or 72 hours. Topical treatment with POPS temperature-sensitive hydrogel extracts significantly reduced erythema, edema, and erosion symptoms (n=6 per group). (D-F) Quantitative severity scoring of the dorsal skin of C57BL/6J mice post-treatment; scoring details are consistent with those in Figure S1. (G) Representative hematoxylin and eosin (HE)-stained images of C57BL/6J mice from each group. (H) Quantitative analysis of epidermal thickness measured by HE staining. ***p<0.001; **p<0.01; *p<0.05 vs. CON; ###p<0.001; ##p<0.01; #p<0.05 vs. MOD.

The selection of thermosensitive hydrogels as POPS extract carriers was based on their multiple advantageous properties, including controlled release kinetics, enhanced skin permeation, and precise dosing capabilities. Most notably, their distinctive phase transition characteristic—maintaining a liquid state at room temperature while forming a gel at physiological temperature—enables convenient application and optimizes active compound retention. This delivery system not only enhances experimental standardization but also closely replicates clinical topical application methods. To evaluate POPS extracts' therapeutic efficacy, we established five experimental groups: control, model, positive control drug (HBC), thermosensitive hydrogel, and POPS treatment groups (at concentrations of 10%, 20%, and 40%). Following UV exposure, the model group exhibited characteristic solar dermatitis symptoms, including increased scratching behavior **(Figure 2B)**, pronounced erythema, and significant skin edema** (Figure 2C)**. Detailed histological examination through H&E staining revealed marked epidermal thickening and extensive dermal inflammatory cell infiltration **(Figures 2G, H)**. Treatment with POPS extract-loaded temperature-sensitive *in situ* hydrogels resulted in significant symptom reduction, with increasing concentrations (10%, 20%, and 40%) showing dose-dependent improvements in skin condition** (Figures 2D-F)**. Notably, the 40% POPS formulation demonstrated therapeutic efficacy comparable to HBC, effectively reducing inflammation without inducing the epidermal thinning observed in the HBC-treated group. The hydrogel delivery system facilitated controlled and sustained POPS release, thereby extending its protective effects. Importantly, the thermosensitive hydrogel control group showed no significant therapeutic benefit, confirming that the observed protective effects were specifically attributable to the POPS extracts. These results demonstrate that POPS extracts effectively attenuate UV-induced skin damage in our solar dermatitis model, with efficacy exhibiting a clear dose-dependent relationship.

### Pharmacological activity analysis of POPS extracts

**Figure 3:**
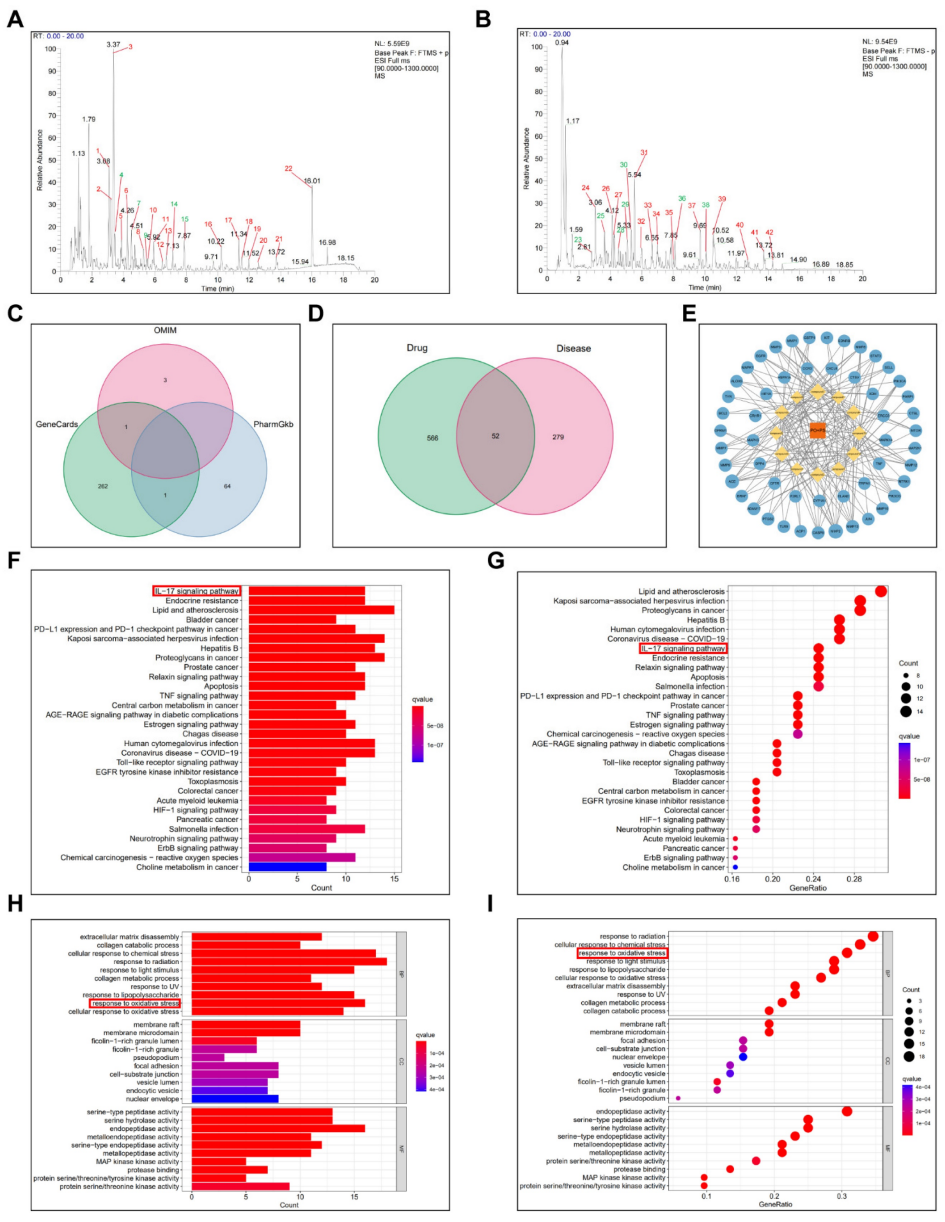
** Identification of pharmacological components of POPS and their network pharmacological analysis for the treatment of solar dermatitis.** (A) Base peak chromatogram (BPC) of POPS in positive ion mode. (B) BPC of POPS in negative ion mode. The green color indicates the 12 pharmacological components. (C) Venn diagram of the solar dermatitis datasets from the GeneCards, OMIM, and PharmGkb gene databases. (D) Venn diagram showing the common targets of the 12 pharmacological components and disease targets of solar dermatitis. (E) POPS-pharmacological component-target gene network diagram. POPS is represented in red, the 12 pharmacological components are represented in yellow, and the targets are represented in blue. (F) Histogram of Kyoto Encyclopedia of Genes and Genomes (KEGG) pathway enrichment analysis of key shared genes. (G) Bubble chart of the KEGG pathway enrichment analysis of key shared genes. (H) Histogram of the results of the Gene Ontology (GO) enrichment analysis of 52 key shared genes. (I) Bubble chart of the GO enrichment analysis of key shared genes.

We employed UPLC-HRMS analysis to investigate the pharmacologically active constituents of POPS, comparing base peak chromatograms (BPCs) in both positive and negative ionization modes **(Figures S2C, D)**. The analysis revealed substantial differences between herbal and biological samples, as well as between the POPS (40%)-treated and untreated model groups. Compound identification was performed using database searches with stringent criteria: mass errors below 25 ppm and fragmentation spectra matching scores exceeding 0.7. The analysis identified 42 distinct chromatographic peaks** (Figures 3A, B)**, with 12 peaks corresponding to administered compounds** (Figure S2E)**. In the medicated group, these peaks showed intensities more than threefold higher than those in the control group, with comprehensive compound information detailed in **Table 2**.

To elucidate the therapeutic mechanisms, we employed network pharmacology analysis, an innovative integrated "drug-target-disease" analytical approach that facilitates understanding of the pharmacological mechanisms of TCM formulas. This widely-adopted methodology enables comprehensive investigation of TCM disease targets and mechanisms. Through network pharmacology analysis, we investigated the protective mechanisms of POPS extracts' pharmacological components against solar dermatitis. By utilizing Venn diagram analysis to identify the intersection between the therapeutic targets of 12 pharmacological components and solar dermatitis disease targets, we identified 52 potential therapeutic targets** (Figure 3D)**. These findings were visualized through both a PPI network **(Figure S5)** and a "TCM-Pharmacological Compound-Action Target" network using Cytoscape 3.8.0 **(Figure 3E)**. KEGG pathway analysis revealed that POPS's therapeutic effects on solar dermatitis involve multiple inflammation-related signaling pathways, with particular emphasis on the IL-17 and TNF signaling pathways **(Figures 3F, G).**

**Figure 4:**
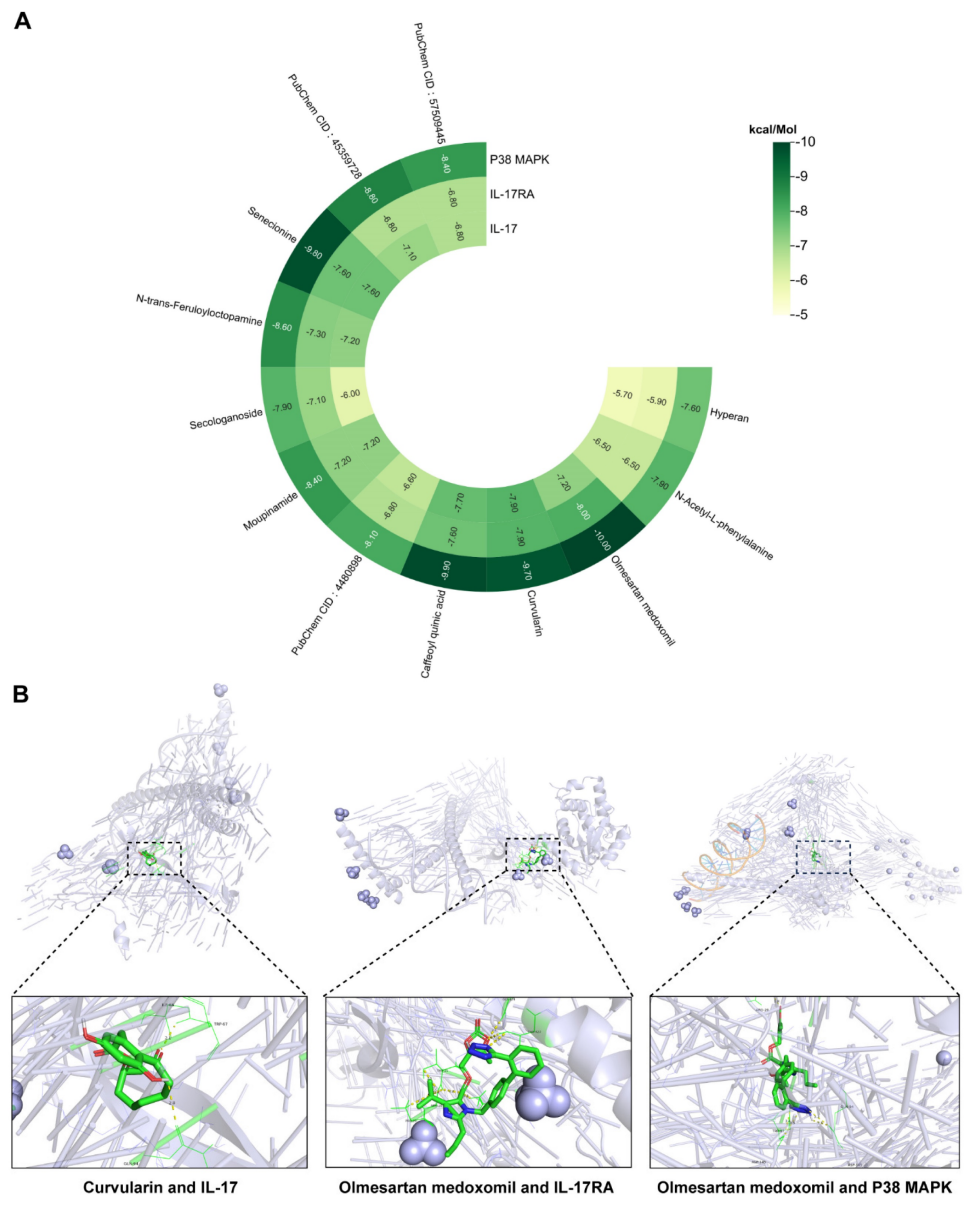
** Heatmap and visualization of the binding of pharmacological components in POPS to molecules of key proteins of the IL-17 pathway.** (A) Heatmap showing the binding affinity of 12 pharmacological components for the IL-17, IL-17RA, and p38 MAPK proteins. (B) Three-dimensional molecular docking conformations of curvularin with the IL-17 protein and of olmesartan medoxomil with the IL-17RA and p38 MAPK proteins.

Notably, IL-17 signaling pathway activation has been strongly implicated in disease progression 23. IL-17 (specifically IL-17A), a proinflammatory factor produced by epithelial and immune cells, plays a crucial role in regulating immune and inflammatory responses. In solar dermatitis patients, elevated IL-17 expression induces the release of additional proinflammatory and chemotactic factors, thereby amplifying the inflammatory cascade 16. UVB irradiation triggers immune cell activation and subsequent IL-17 production, leading to enhanced infiltration of neutrophils and macrophages, ultimately exacerbating skin inflammation 17. Structurally, IL-17R comprises five subunits, with IL-17RA serving as a critical target for blocking IL-17-mediated inflammation 24. Additionally, p38 MAPK, a protein kinase activated by proinflammatory and environmental stressors, serves as a crucial regulator of various biological processes, including inflammation 25, primarily through its ability to phosphorylate and activate c-Jun 26. Molecular docking analyses of the 12 identified compounds with IL-17 pathway targets revealed significant interactions, particularly between curvularin and IL-17 receptors, and between olmesartan medoxomil and both IL-17RA and p38 MAPK **(Figure 4A)**. The binding energy calculations yielded the following results: curvularin/IL-17 (-9.7 kcal/mol), olmesartan medoxomil/IL-17RA (-8.0 kcal/mol), and olmesartan medoxomil/p38 MAPK (-10.0 kcal/mol) **(Figures 4A, B)**, providing strong evidence for the potential therapeutic mechanism via the IL-17/IL-17RA/p38 MAPK signaling pathway.

### POPS extracts alleviate solar dermatitis via IL-17 pathway inhibition in mice

**Figure 5:**
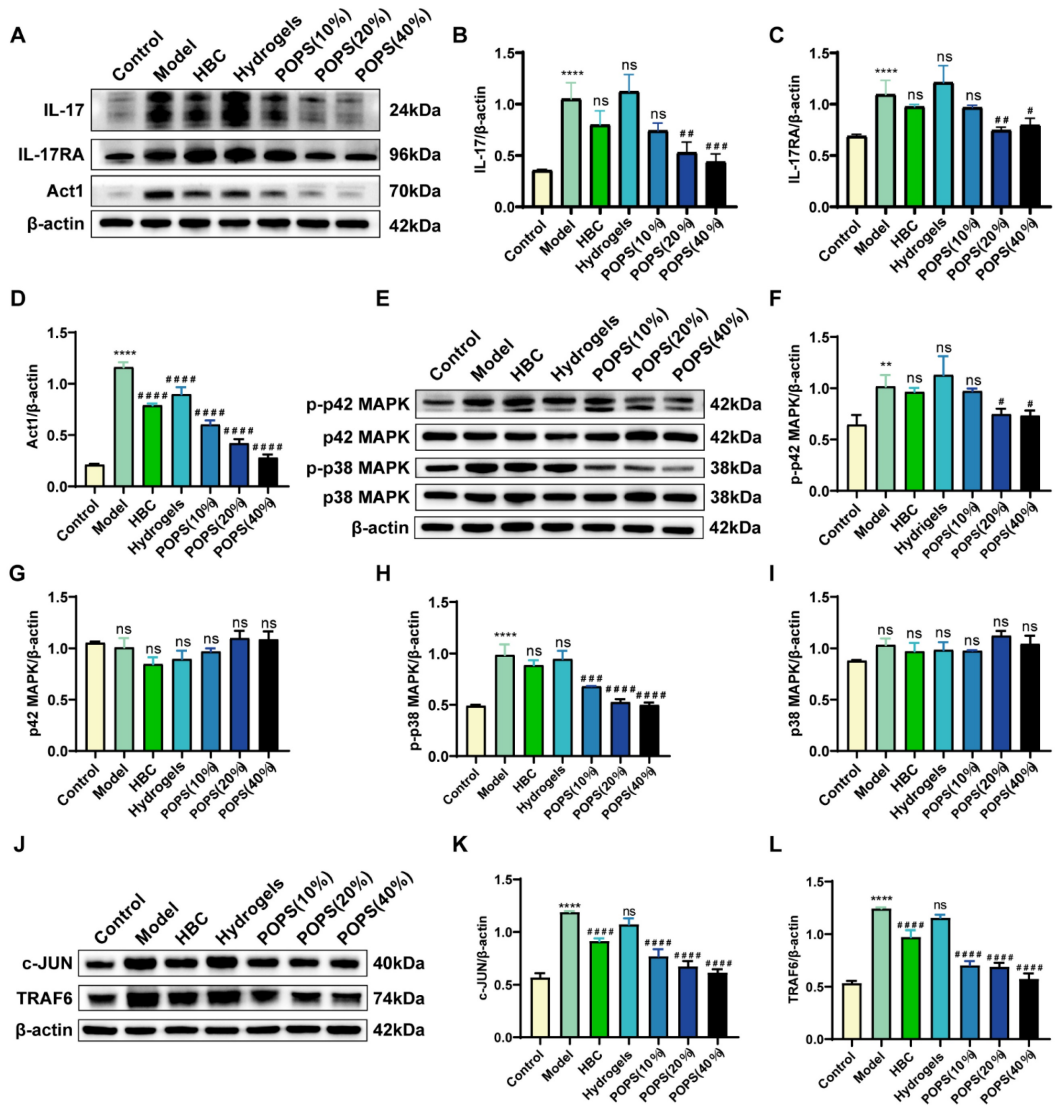
** POPS extracts alleviate solar dermatitis via inhibition of the IL-17 pathway in mice.** (A) Western blot analysis of the protein expression levels of IL-17A, IL-17RA, and Act1 in each group. (B-D) Quantitative analysis of the Western blot results for IL-17A, IL-17RA, and Act1 (n=3 per group). (E) Western blot analysis of p42 MAPK, p-p42 MAPK, p38 MAPK, and p-p38 MAPK protein expression levels in each group. (F-I) Results from the quantitative analysis of the Western blot results for p42 MAPK, p-p42 MAPK, p38 MAPK, and p-p38 MAPK (n=3 per group). (J) Western blot analysis of TRAF6 and c-JUN protein expression levels in each group. (K-L) Quantitative analysis of the Western blot results for TRAF6 and c-JUN (n=3 per group). ***p<0.001; **p<0.01; *p<0.05 vs. CON; ###p<0.001; ##p<0.01; #p<0.05 vs. MOD.

The activation of the IL-17 signaling pathway triggers multiple pathological changes, including stratum corneum thickening, epidermal layer abnormalities, and keratinocyte apoptosis, ultimately leading to inflammatory factor release and solar dermatitis development 27, 28. Based on these pathological mechanisms, IL-17 pathway inhibition has emerged as a promising therapeutic strategy. To validate our network pharmacology findings from KEGG pathway enrichment analysis, we investigated POPS extracts' capacity to ameliorate solar dermatitis through IL-17 signaling pathway modulation. We employed Western blot analysis to quantitatively assess the expression levels of key pathway proteins. Notably, our results demonstrated marked upregulation of the IL-17, IL-17RA, and ACT1 proteins in the model group compared to the control group. Most importantly, POPS treatment significantly reduced the expression of these proteins **(Figure 5A-D)**, demonstrating its modulatory effect on the IL-17 pathway. Further investigation of downstream effector molecules revealed that POPS treatment suppressed both p38 MAPK and p42 MAPK expression, with a particularly notable inhibition of p38 phosphorylation compared to p42 phosphorylation **(Figure 5E-I)**. These observations suggest that POPS's therapeutic efficacy is predominantly mediated through p38 activation inhibition. In line with these findings, POPS treatment effectively downregulated the expression of TRAF6, which serves as a key signaling mediator in the IL-17 cascade, and the transcription factor c-JUN in solar dermatitis skin samples **(Figure 5J-L)**. Collectively, these experimental data provide robust evidence that POPS significantly attenuates the UVB-induced activation of the IL-17/IL-17RA/p38 MAPK signaling cascade. This comprehensive inhibitory effect on multiple pathway components elucidates the molecular mechanism underlying POPS's therapeutic efficacy in solar dermatitis treatment.

### POPS extracts reduce macrophage infiltration and alleviate inflammation via IL-17/CCL2 pathway inhibition

**Figure 6:**
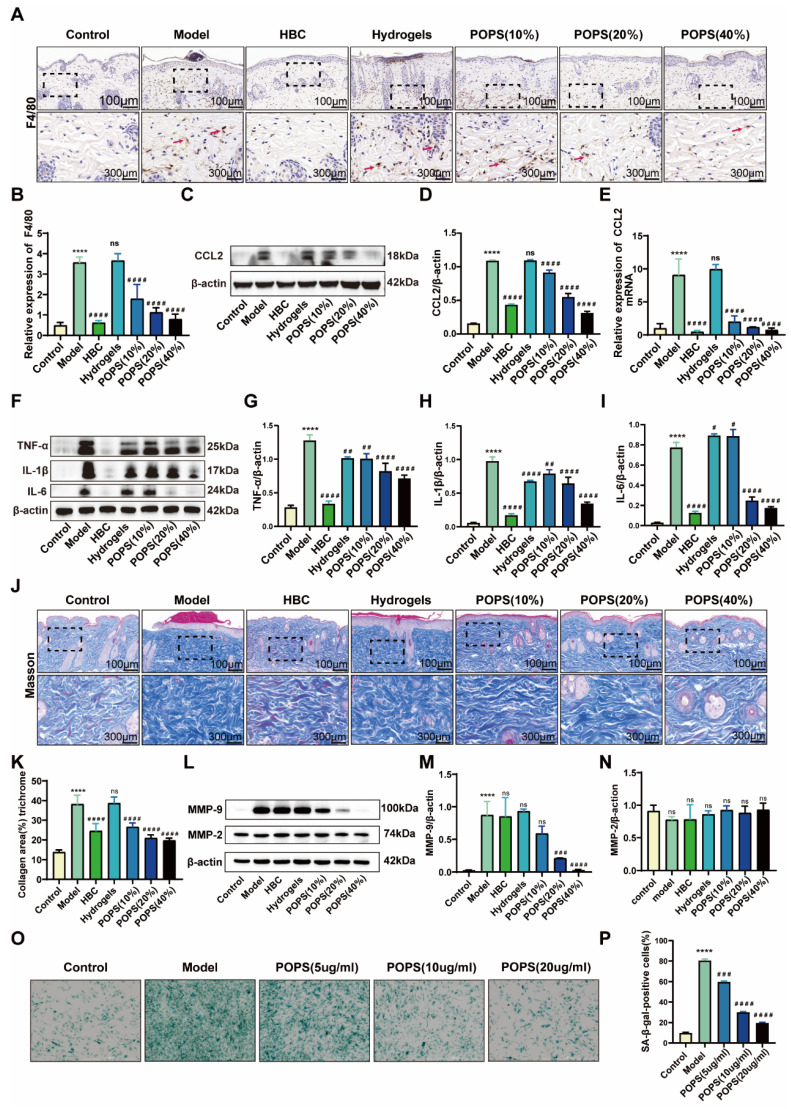
** POPS extracts reduce macrophage infiltration and alleviate inflammation via inhibition of the IL-17/CCL2 pathway.** (A) Representative images of F4/80 immunohistochemical staining of the skin of C57BL/6J mice 72 hours post-treatment. (B) Quantitative analysis of F4/80 immunohistochemical staining. (C) Western blot analysis of CCL2 protein expression levels in each group. (D) Quantitative analysis of the Western blot results for CCL2 (n=3 per group). (E) qRT‒PCR analysis of CCL2 mRNA expression levels in each group (n=3 per group). (F) Western blot analysis of IL-1β, IL-6, and TNF-α protein expression levels in each group. (G-I) Results from the quantitative analysis of the Western blot results for IL-1β, IL-6, and TNF-α (n=3 per group). (J-K) Quantitative analysis of collagen fiber abundance via Masson's trichrome staining. (L-N) Western blot analysis and quantitative assessment of MMP-2 and MMP-9 protein expression levels in each group. (O) Representative images illustrating the impact of macrophages on the level of inflammaging in fibroblasts following POPS intervention at various concentrations were obtained via SA-β-gal staining. (P) Quantitative analysis of SA-β-gal-positive cells measured by SA-β-gal staining. All data are presented as the means ± SDs. Statistical significance: ***p<0.001; **p<0.01; *p<0.05 vs. CON; ###p<0.001; ##p<0.01; #p<0.05 vs. MOD.

Initial histological examination through H&E staining revealed pronounced inflammatory cell infiltration in the dermis of the model group **(Figure 2G)**. To characterize the inflammatory response in detail, we performed immunohistochemical analyses, specifically quantifying F4/80+ macrophage infiltration in the affected tissues. The analysis revealed a significant increase in macrophage populations within the model group, while the POPS-treated group demonstrated a marked reduction in infiltrating cells **(Figures 6A, B)**. Notably, these observations align with previous research indicating that IL-17 signaling pathway activation enhances macrophage recruitment through CCL2-mediated chemotaxis, subsequently intensifying skin inflammation 29, 30. To validate this mechanism, we conducted comprehensive analyses of CCL2 expression using both Western blotting and qRT‒PCR techniques. The results demonstrated significantly elevated CCL2 protein expression in the model group, with a substantial reduction observed in the POPS-treated group (**Figures 6C-E**)**.** To further characterize the inflammatory response, we evaluated the expression profiles of key macrophage-associated inflammatory cytokines, including IL-1β, IL-6, and TNF-α, through parallel analyses using Western blotting** (Figures 6F-I)** and qRT‒PCR **(Figures S3A‒C)**. The results revealed a dose-dependent reduction in these inflammatory mediators across the treatment groups, with higher POPS concentrations yielding more pronounced suppression of cytokine expression. These comprehensive findings demonstrate that POPS extract effectively attenuates both macrophage infiltration and inflammatory responses through targeted inhibition of the IL-17/CCL2 signaling axis.

We assessed collagen fiber distribution and morphology using Masson's trichrome staining. The analysis revealed pathological collagen fiber proliferation in the model group, which was significantly attenuated in the POPS-treated group **(Figures 6J, K)**. Importantly, this observation aligns with previous studies demonstrating that UV-induced premature skin aging correlates with upregulation of matrix metalloproteinases (MMPs), particularly MMP-2 and MMP-9, these collagen-degrading enzymes whose expression increases during inflammation responses 31, 32. To further investigate this mechanism, we analyzed the protein expression profiles of MMP-2 and MMP-9 via Western blotting **(Figures 6L-N).** The results demonstrated that POPS extract specifically suppressed the elevated MMP-9 expression observed in the model group, while MMP-2 expression remained relatively constant across all experimental groups. These findings suggest selective regulation of specific MMPs by POPS extract. Previous research has established that increased macrophage infiltration in skin tissue can accelerate inflammation-driven aging processes 33. Based on our observations, we hypothesized that the pathological alterations in collagen fiber structure within the model group were primarily mediated through macrophage-derived MMP-9. To test this hypothesis, we examined the impact of POPS extract treatment on macrophage-induced β-galactosidase activity in fibroblasts **(Figures 6O, P)**. Fibroblast β-galactosidase activity showed significant elevation when co-cultured with UVB-induced macrophages, strongly suggesting that UVB-triggered macrophage recruitment promotes inflammaging. Notably, POPS extract treatment significantly reduced this β-galactosidase activity, demonstrating its ability to suppress macrophage-induced inflammatory senescence in fibroblasts. Collectively, these findings demonstrate that POPS extract exhibits dual therapeutic effects: first, suppressing macrophage-induced inflammation, and second, inhibiting macrophage-mediated inflammaging through modulation of the IL-17/CCL2 pathway.

### POPS extracts reduce sunburn cell production by ameliorating UVB-induced oxidative stress

**Figure 7:**
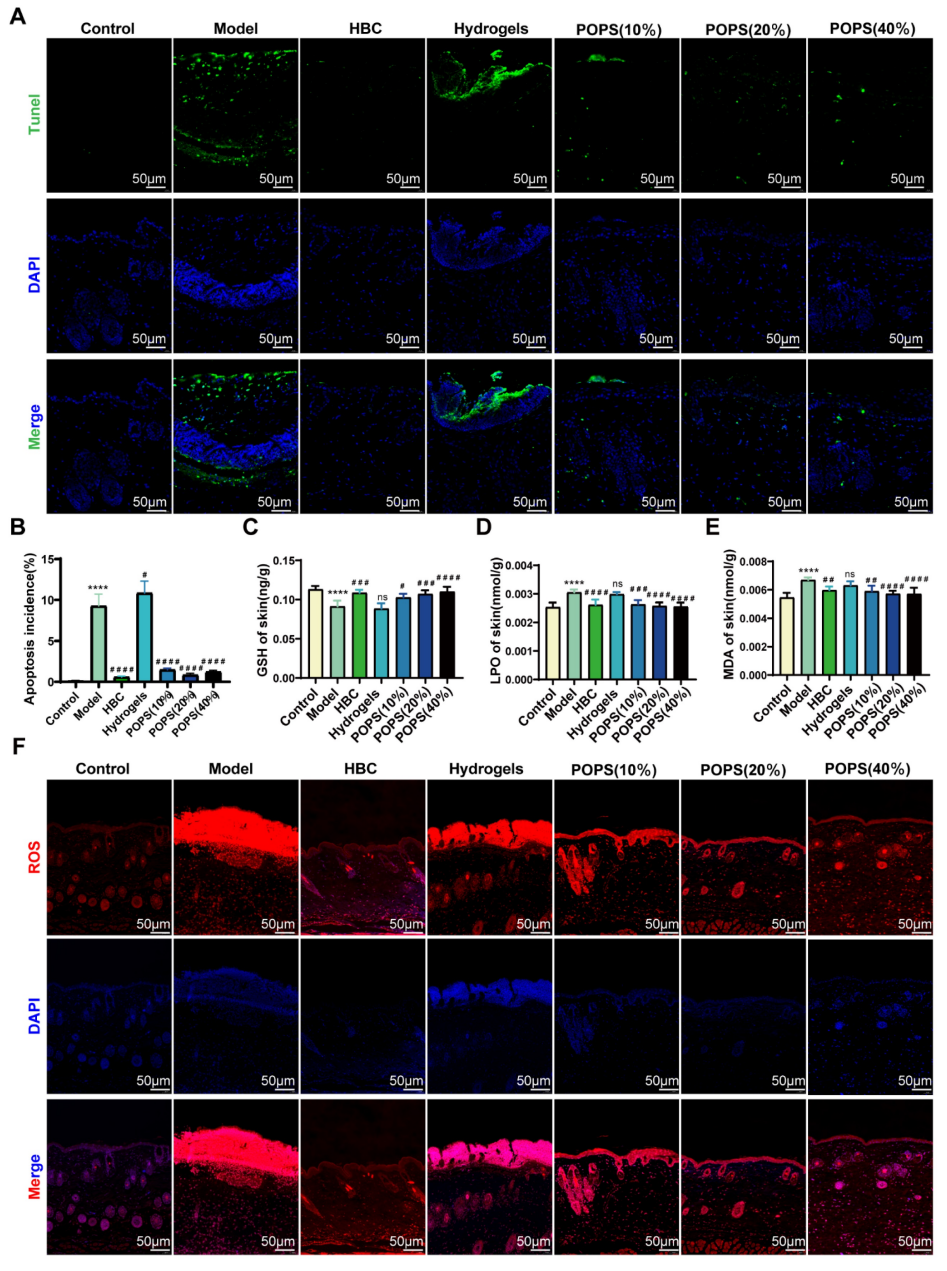
** POPS extracts reduce sunburn cell production by ameliorating UVB-induced oxidative stress.** (A-B) Immunofluorescence staining and quantitative analysis of TUNEL (green) staining of skin tissues from each group. (C-E) ELISA analysis of GSH, LPO, and MDA levels in the skin tissues of C57BL/6J mice (n=6 per group). (F) Immunofluorescence staining of ROS (red) in the skin tissues of each group. All the data are presented as the means ± SDs. Statistical significance: ***p<0.001; **p<0.01; *p<0.05 vs. CON; ###p<0.001; ##p<0.01; #p<0.05 vs. MOD.

Sunburn cells (SBCs), characterized as apoptotic keratinocytes, emerge as a consequence of UV radiation-induced oxidative stress 34. UV exposure triggers increased reactive oxygen species (ROS) production, resulting in DNA damage and inflammation, ultimately compromising skin barrier integrity and initiating solar dermatitis 35. Our GO analysis revealed that solar dermatitis involves multiple biological processes, including responses to oxidative stress, ROS, and UV radiation, along with regulation of apoptotic signaling pathways **(Figures 3H, I)**. Building upon GO enrichment analysis results demonstrating that POPS's therapeutic effects correlate with oxidative stress response regulation, We investigated POPS extract's potential to ameliorate UVB-induced oxidative stress in skin tissue. Using immunofluorescence TUNEL assays, we evaluated UVB-induced apoptosis and observed significant apoptotic activity in the model group, which was markedly suppressed in POPS-treated specimens **(Figure 7A, B)**. To comprehensively assess UVB-induced oxidative stress, we employed ELISA methodology to quantify specific oxidation products: malondialdehyde (MDA), lipid peroxides (LPO), and the antioxidant glutathione (GSH). The analysis revealed that POPS treatment effectively reduced MDA and LPO levels in UVB-exposed skin tissues while simultaneously enhancing GSH levels** (Figures 7C-E)**. These findings indicate significant antioxidant activity of POPS extract. Previous research has established that excessive ROS production leads to cellular damage and inflammatory responses 36. Our results demonstrate that POPS significantly attenuates UVB-induced ROS production in skin tissue, with particularly pronounced effects on epidermal keratinocytes **(Figures 7F and S4)**. These comprehensive establish that POPS extracts effectively counteract UV-induced oxidative stress in skin tissue, consequently protecting keratinocytes from UV-induced apoptosis and reducing SBC formation. This protective mechanism represents a crucial component of POPS's therapeutic efficacy against solar dermatitis.

## Conclusions

In this study, we investigated the therapeutic potential of POPS extracts for treating UVB-induced solar dermatitis in a mouse model. The POPS significantly alleviated skin inflammation, reduced macrophage infiltration, and suppressed macrophage-associated inflammatory responses. These effects were attributed to the inhibition of the IL-17/CCL2 signaling pathway and the reduction of oxidative stress, which collectively delayed keratinocyte apoptosis. Network pharmacology analysis of the 12 pharmacologically active components in POPS suggested their involvement in regulating oxidative stress and modulating the IL-17 pathway. Macrophage infiltration in the dermis was significantly suppressed, accompanied by a marked attenuation of macrophage-associated inflammaging, thereby elucidating POPS's dual efficacy in modulating both inflammatory and oxidative cascades. Despite these promising outcomes, several limitations remain. Although we identified 12 active components, the specific compounds responsible for the observed effects require further isolation and validation. Additionally, other molecular mechanisms beyond the IL-17/CCL2 pathway remain unexplored and warrant broader investigation into alternative pathways. Furthermore, since this study utilized a specific formulation of POPS in temperature-sensitive hydrogels, further optimization across different formulations and concentrations is needed to enhance its clinical relevance. In conclusion, POPS demonstrates strong potential as a therapeutic approach for UVB-induced skin injury and other inflammatory skin conditions by regulating IL-17/CCL2 signaling and oxidative stress. However, further research is necessary to refine its formulation and identify additional therapeutic targets.

## Supplementary Material

Supplementary figures.

## Figures and Tables

**Table 1:** Primers used for quantitative PCR.

Target gene	Forward primer (5′ -3′)	Reverse primer (5′ -3′)
GAPDH	CCTCGTCCCGTAGACAAAATG	TGAGGTCAATGAAGGGGTCGT
IL-1β	GCAGTGGTTCGAGGCCTAAT	GCTGCTTCAGACACTTGCAC
IL-6	CTTCTTGGGACTGATGCTGGTGAC	TCTGTTGGGAGTGGTATCCTCTGTG
TNF-α	ACCCTCACACTCACAAACCAC	ACAAGGTACAACCCATCGGC
CCL2	CACTCACCTGCTGCTACTCA	GAGCTTGGTGACAAAAACTACAGC

**Table 2:** Identification of POPS pharmacological components in skin using UPLC-HRMS.

No.	Compound	PubChem CID	m/z	RT min	ppm	Adduct	Score	Source
1	Hyperan	3316	222.1489	3.43	0.3	[M+H]+	0.7141	*Portulaca oleracea*
2	Senecionine	25244787	308.1857	4.5	0.7	[M+H-CO]+	0.888	*Portulaca oleracea*
3	N-trans-Feruloyloctopamine	24096391	312.1229	5.44	0.3	[M+H-H2O]+	0.9883	*Portulaca oleracea*
4	Moupinamide	5280537	314.1386	7.12	2.2	[M+H]+	0.9417	*Portulaca oleracea and Patrinia scabiosifolia*
5	(Z)-2,6-dimethyl-7-(4- methyl-5-oxooxolan-2-yl)- 3-[[3,4,5-trihydroxy-6- (hydroxymethyl)oxan-2- yl]oxymethyl]hept-5-enoic acid	45359728	469.2042	7.85	1.3	[M+Na]+	0.9959	*Patrinia scabiosifolia*
6	Caffeoyl quinic acid	348159	353.0879	2.81	2.9	[M-H]-	0.9951	*Portulaca oleracea and Patrinia scabiosifolia*
7	Secologanoside	14136853	389.109	3.65	0.2	[M-H]-	0.9311	*Patrinia scabiosifolia*
8	CHEBI:139442	4480898	387.1664	4.53	1.1	[M-H]-	0.9586	*Portulaca oleracea*
9	N-Acetyl-L-phenylalanine	74839	206.0825	5.1	0.6	[M-H]-	0.9686	*Portulaca oleracea and Patrinia scabiosifolia*
10	6-(3-(.beta.-D-Glucopyranosyloxy)butyl)- 5,5-dimethyl-3- oxocyclohex-1-ene-1- carboxylic acid	57509445	401.1822	5.33	0.2	[M-H]-	0.9947	*Portulaca oleracea*
11	Olmesartan medoxomil	130881	557.2245	8.1	16.4	[M-H]-	0.8502	*Patrinia scabiosifolia*
12	Curvularin	296197	291.124	10.08	0.4	[M-H]-	0.9984	*Patrinia scabiosifolia*

## Abbreviations

BPCs: base peak chromatograms
BP: biological processes
ELISA: enzyme-linked immunosorbent assay
GO: Gene Ontology
HE: hematoxylin-eosin
KEGG: Kyoto Encyclopedia of Genes and Genomes
LPO: lipid peroxides
MDA: malondialdehyde
NO: nitric oxide
HBC: positive control drug
PBS: phosphate-buffered saline
POPS: Portulaca oleracea and Patrinia scabiosaefolia
PPI network: protein-protein interaction network
qRT‒PCR: quantitative reverse transcription polymerase chain reaction
ROS: reactive oxygen species
SA-β-gal: senescence-associated β-galactosidase
SBCs: sunburn cells
TCM: traditional Chinese medicine
UV: ultraviolet
UVB: ultraviolet B
UPLC-HRMS: ultra-performance liquid chromatography-high resolution mass spectrometry
GSH: glutathione
