# Supplementary material for: Extracts of Portulaca oleracea and Patrinia scabiosaefolia relieve ultraviolet B-induced skin injury in solar dermatitis mice via inhibiting IL-17/CCL2 pathway and oxidative stress
Source: Int J Med Sci. 2025 Jan 21;22(4):856–72. doi: 10.7150/ijms.106289 (PMC11843150; doi:10.7150/ijms.106289)
Supplement: Supplementary file 1 — Supplementary figures. [file ijmsv22p0856s1.pdf]

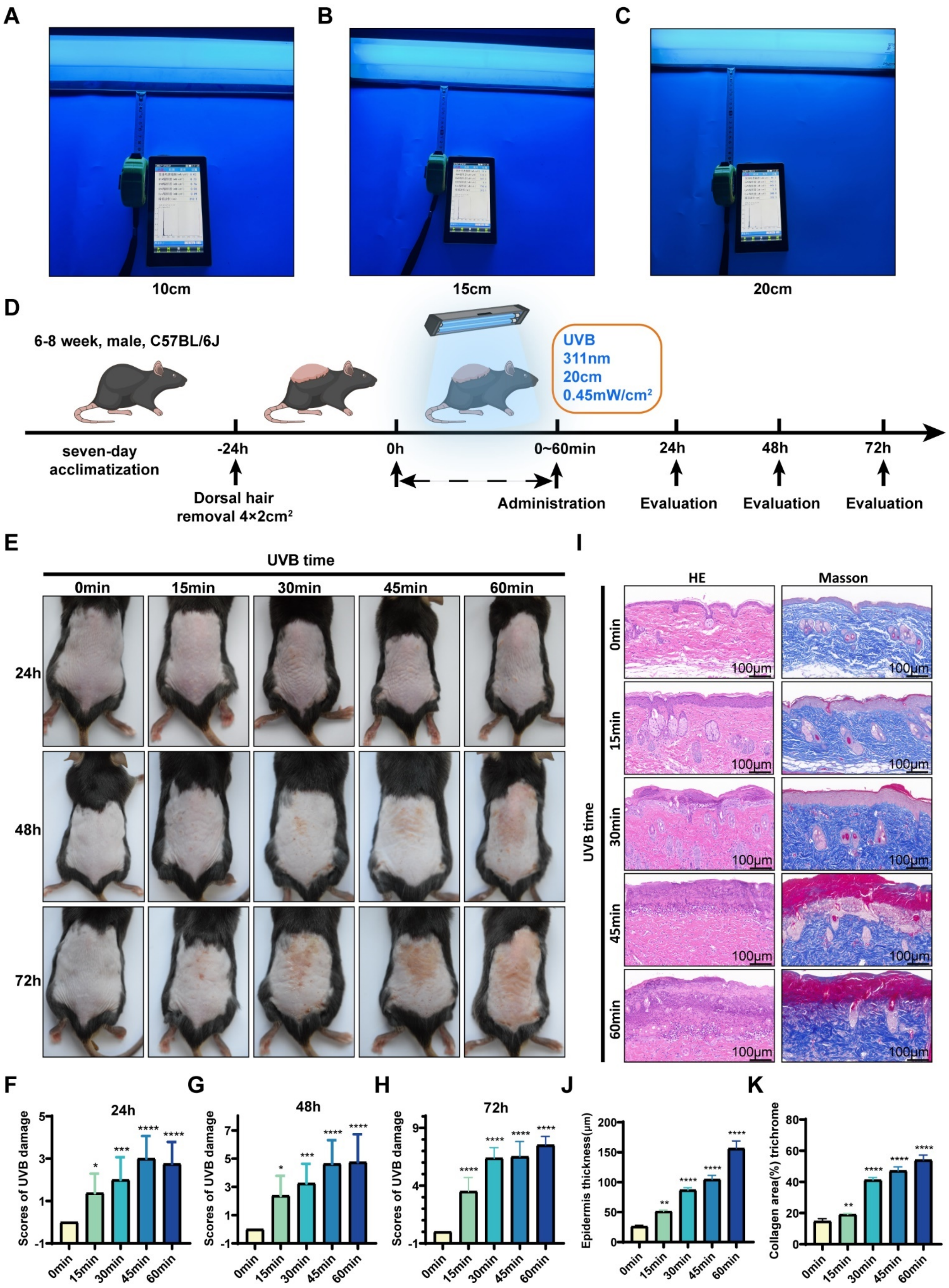

Figure S1. Raw images of UV intensity measurements at different distances and establishment of a solar dermatitis mouse model. (A-C) Measurements of UVA, UVB, and UVC irradiance and peak wavelength (nm) intensity of ultraviolet lamps at irradiation distances of 10, 15, and 20 cm, respectively. (D) Schematic diagram illustrating UVB irradiation of the dorsal skin of C57BL/6J mice. Mice were exposed to UVB for 0, 15, 30, 45, or 60 minutes. Changes in skin appearance were recorded post-irradiation, and histopathological analysis was performed 72 hours later. (E) Representative photographs showing changes in the appearance of C57BL/6J mice at 24, 48, and 72 hours (n=8 per group). (F-H) Quantitative severity scoring of the dorsal skin of C57BL/6J mice at 24, 48, and 72 hours post-UVB irradiation. Scores were calculated as the sum of individual item scores, with detailed scoring criteria provided in the "Materials and Methods" section. (I) Representative images of hematoxylin and eosin (HE) and Masson's trichrome staining in mouse groups 72 hours post-UVB irradiation. (J) Quantitative analysis of epidermal thickness measured by HE staining. (K) Quantitative analysis of the collagen fiber ratio. ***p<0.001; **p<0.01; *p<0.05 compared to the 0-minute group.

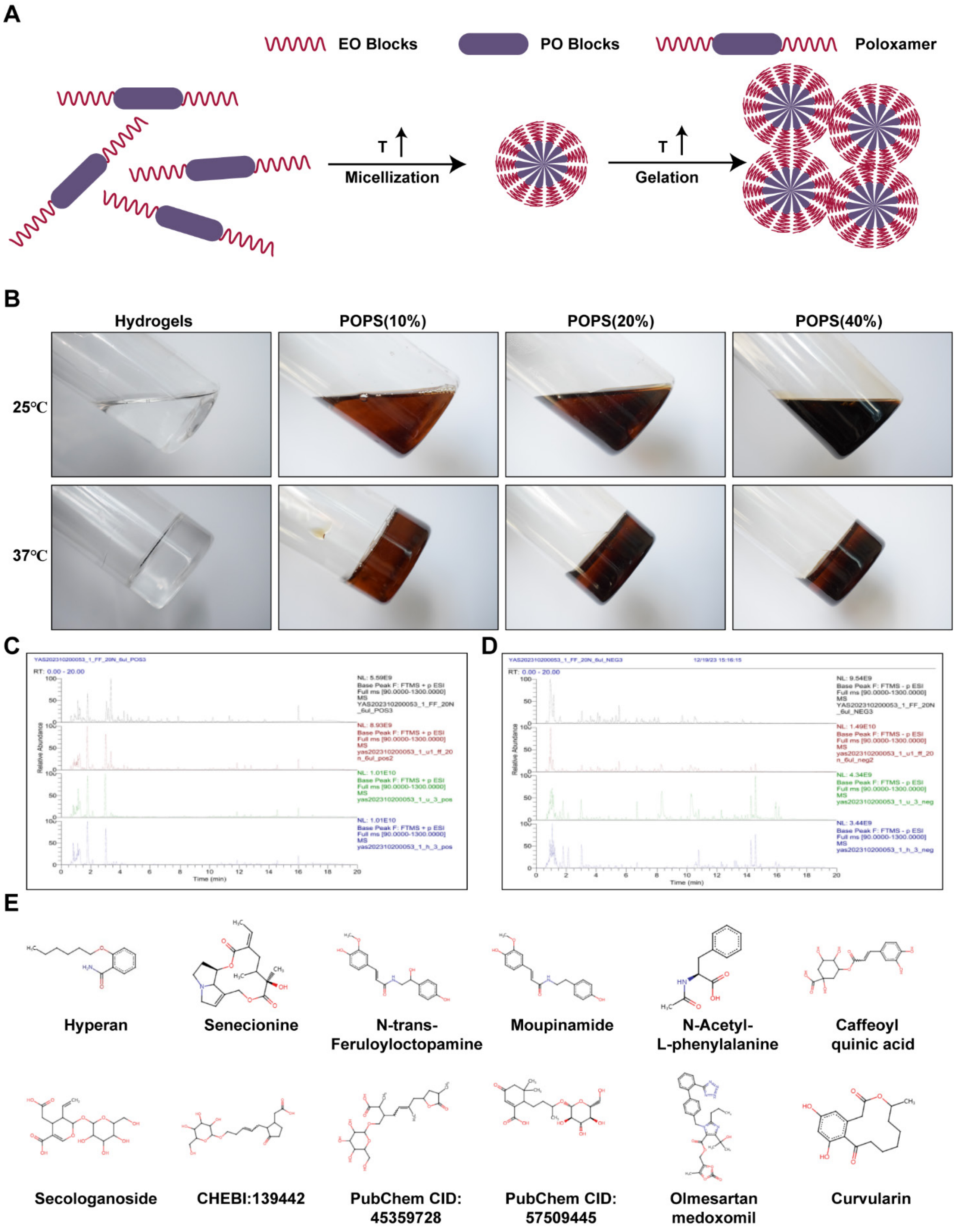

Figure S2. Preparation of POPS temperature-sensitive in situ hydrogels and identification of the pharmacological components of POPS. (A) Schematic diagram of the thermosensitive Poloxamer hydrogel. (B) Thermosensitive hydrogel matrix and POPS at low, medium, and high concentrations in the liquid and solid states at 25°C and 37°C, respectively. (C) Base peak chromatograms (BPCs) of samples in positive ion mode. (D) BPC of samples in negative ion mode. The spectra are arranged from top to bottom as follows: the POPS sample, the model group + POPS sample, the model group, and the POPS (40%) treatment group. (E) Chemical structures of the 12 pharmacological compounds.

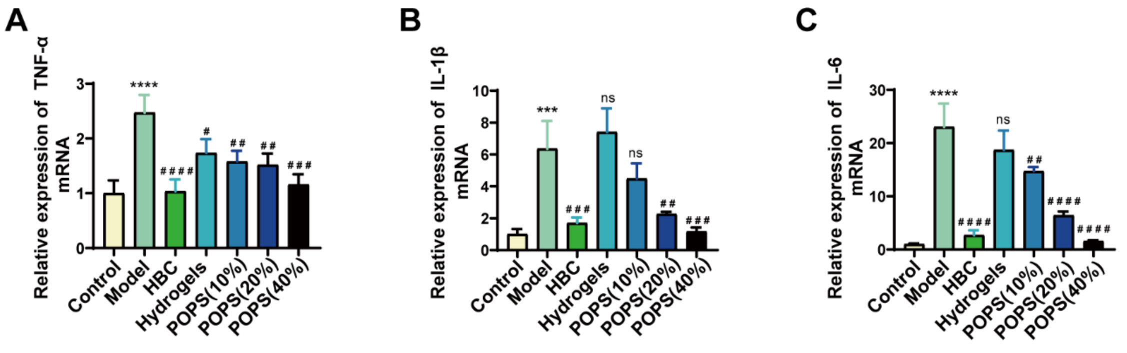

Figure S3. POPS extracts reduce macrophage infiltration and alleviate related inflammaging. (A-C) qRT–PCR analysis of IL-1β, IL-6, and TNF-α mRNA expression levels in each group (n=3 per group). All the data are presented as the means ± SDs. Statistical significance: ***p<0.001; **p<0.01; *p<0.05 vs. CON; ###p<0.001; ##p<0.01; #p<0.05 vs. MOD.

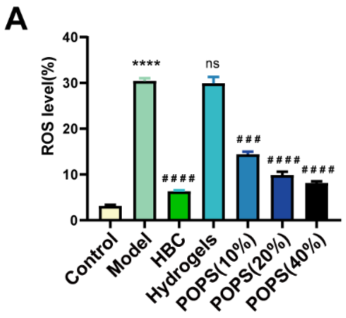

Figure S4. POPS extracts reduce sunburn cell production by ameliorating UVB-induced oxidative stress. (A) Quantitative analysis of ROS (red) immunofluorescence staining of skin tissues from each group. All the data are presented as the means ± SDs. Statistical significance: ***p<0.001; **p<0.01; *p<0.05 vs. CON; ###p<0.001; ##p<0.01; #p<0.05 vs. MOD.

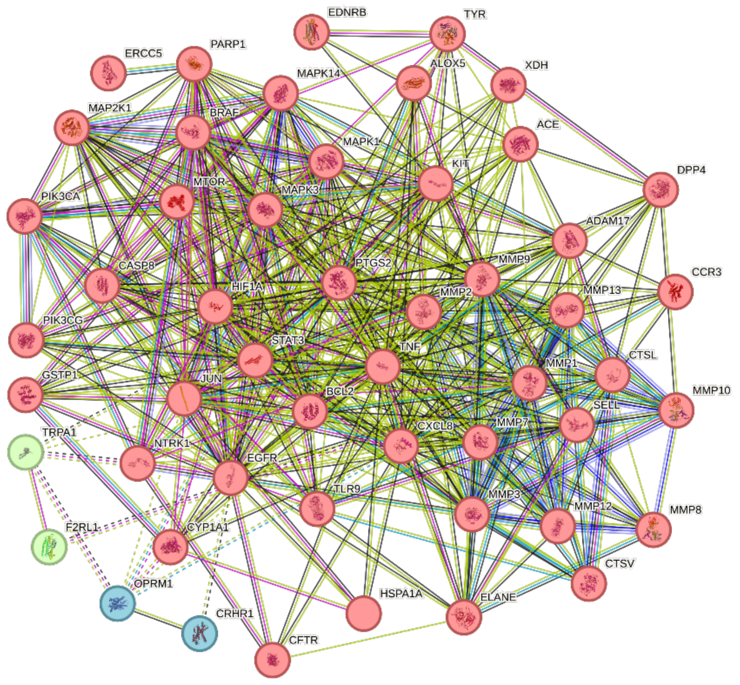

Figure S5. Construction of a PPI network for 52 key intersecting genes via STRING.
